# Evidence for Continuing Professional Development and Recency of Practice Standards for Regulated Health Professionals in Australia: Protocol for a Systematic Review

**DOI:** 10.2196/28625

**Published:** 2022-04-13

**Authors:** Penelope Main, Sarah Anderson

**Affiliations:** 1 Research and Evaluation Team Australian Health Practitioner Regulation Agency Adelaide Australia; 2 Research and Evaluation Team Australian Health Practitioner Regulation Agency Melbourne Australia; 3 Department of Prosthetics and Orthotics LaTrobe University Bundoora Australia

**Keywords:** protocol, systematic review, continuing professional development, continuing education, recency of practice, regulatory standards, health practitioners

## Abstract

**Background:**

Continuing professional development (CPD) and recency of practice (ROP) standards are components of health practitioner regulation in Australia. The CPD and ROP standards are currently under review, and an evidence base to assist the development of consistent standards is required. Preliminary searching was unable to find a recent systematic review of the literature to provide an evidence base to underpin the standards review.

**Objective:**

This paper presents the protocol for a systematic review that aims to develop a current evidence base that will support the National Boards to develop more consistent, evidence-based, effective standards that are clear and easy to understand and operationalize.

**Methods:**

Research questions were developed to support the planned review of CPD and ROP registration standards. Major databases and relevant journals were searched for articles published in English between 2015 and 2021, using key search terms based on previous unpublished reviews of the CPD and ROP registration standards. The quality of the articles retrieved will be assessed using an instrument suitable for use in the development of public policy. The findings will be published in a peer-reviewed journal.

**Results:**

In September 2021, our search strategy identified 18,002 studies for the CPD-related research questions after removal of duplicates. Of these, 509 records were screened based on their title, and 66 full-text articles were assessed for eligibility based on their abstract, of which 31 met the inclusion criteria. A further 291 articles were identified as relevant to the ROP research questions. Of these, 87 records were screened based on their title, and 46 full-text articles were assessed for eligibility based on their abstract, of which 8 studies met our inclusion criteria.

**Conclusions:**

This protocol outlines the scope and methodology that will be used to conduct a systematic review of evidence for CPD and ROP and inform a review of the standards for regulated health professionals in Australia. Previous research has shown that while CPD improves practitioner knowledge, the link to public safety is unclear. While there has been a greater focus on maintenance of certification and other quality assurance activities over the past 10 years, there remains great variability in CPD requirements across both professions and jurisdictions. ROP was found to be a poorly researched area with most research concentrating on medical practitioners, nurses, and midwives and no clear consensus about the optimal time period after which retraining or an assessment of competence should be introduced. As the CPD and ROP standards are currently under review, it is timely that a review of current evidence be undertaken.

**International Registered Report Identifier (IRRID):**

DERR1-10.2196/28625

## Introduction

### Background

In July 2010, Australia introduced a national scheme for the regulation of health practitioners [1]. Initially, the National Registration and Accreditation Scheme (National Scheme) regulated 10 health professions (chiropractors, dental practitioners, medical practitioners, nurses and midwives, optometrists, osteopaths, pharmacists, physiotherapists, podiatrists, and psychologists). A further 4 professions (Aboriginal and Torres Strait Islander health practitioners, Chinese medicine practitioners, medical radiation practitioners, and occupational therapists) were brought into the scheme from July 2012, followed by paramedicine in December 2018.

The Health Practitioner Regulation National Law as in force in each state and territory established the Australian Health Practitioner Regulation Agency (Ahpra) to administer the National Scheme, working in partnership with the National Boards for the regulated professions. The National Boards and Ahpra protect the public by regulating health professionals who practice in Australia.

The National Law requires that National Boards must develop, consult on, and recommend certain registration standards to the Australian Health Workforce Ministerial Council. These core registration standards are generally reviewed every 5 years in line with good regulatory practice.

The registration standards for continuing professional development (CPD) and recency of practice (ROP) for health practitioners wishing to renew their registration for most National Boards are currently under review. Aspects of these standards are consistent while others are profession-specific, and there has been a trend toward more consistency over the life of the National Scheme. This systematic review will focus on all health professions regulated by Ahpra to provide an updated evidence base for registration standards for CPD for dental, medical radiation practice, nursing, midwifery, osteopathy, paramedicine, pharmacy, physiotherapy, podiatry, and psychology; and ROP for chiropractic, dental, medical radiation practice, optometry, paramedicine, pharmacy, physiotherapy, podiatry, and psychology.

### Objective

This paper presents a protocol for a systematic review that aims to develop a current evidence base that will support the national boards to develop more consistent, evidence-based, effective standards that are clear and easy to understand and operationalize. It is designed to build on earlier research commissioned and/or undertaken by Ahpra for previous reviews of the CPD and ROP registration standards. The research report will include a summary of findings from earlier reviews and identify new research to provide a comprehensive, contemporary overview of the available evidence on CPD and ROP.

#### Review Questions

The overarching research question for the systematic review is as follows: How can the current registration standard requirements for [insert specific registration standard requirement] for Australian [insert health profession of interest] be as evidence-based and effective as possible in facilitating practitioners to practice safely and competently?

More detailed research questions for the systematic review and international benchmarking study are as follows:

1. What research has been conducted since the previous systematic review in 2015 regarding CPD and ROP for the [insert relevant health professions]?
2. How do the current Australian CPD and ROP standards for the [insert relevant health professions] benchmark against regulators in comparable jurisdictions?

CPD-specific questions are as follows:

1. Is there evidence to support an optimal quantity of CPD to maintain competence? Does the evidence suggest any benefit or disadvantage in requiring CPD to be completed over a particular period such as 1, 2, or 3 years? Is there a case for these to vary between health professions or to vary within the same health profession depending on differences in the scope of practice, practice division, and practice endorsement?
2. Does the evidence indicate that some types of CPD (including virtual) are more effective in improving practitioner competence and patient safety?
3. Is there evidence to suggest whether self-directed CPD or mandated CPD is more effective in promoting practitioners’ competence and patient safety? Should some CPD be mandated? Is a mix of mandated and self-directed CPD more effective? If so, is there an optimal ratio of self-directed to mandated CPD?
4. Is there any evidence that CPD that has been accredited or subject to some quality assurance process is more effective in maintaining clinical competency and/or patient safety outcomes? What factors should be taken into consideration in accrediting CPD?
5. Under what circumstances could an exemption from CPD be justified? Is there evidence to suggest that a short gap in CPD (eg, 1 or 2 years) has a negative effect on professional competency, including any specific time frames for this effect to appear?
6. Is there any evidence to suggest a benefit or disadvantage to requiring CPD that is more focused on maintaining a practitioner’s competence in their current scope of practice? What is the evidence on best practice in supporting CPD for practitioners who may wish to change their scope of practice? Is there any evidence to suggest that CPD contributes to other aspects of professional practice?
7. Is CPD more effective when it is based on a practitioner’s assessment or reflection, and peer review or based on curricula to address their learning needs and skills gap, or is CPD more effective when it is based on meeting an externally set requirement that is measured in hours or points?
8. Should practitioners who hold limited registration (or short-term temporary registration, through the pandemic subregister) be required to undertake CPD?

ROP-specific questions are as follows:

1. With regards to skills retention and skills fade, does the period of time vary between different health professions or at different stages of their career (eg, new graduate, early career, mature or advanced practitioners)?
2. Is there evidence regarding when competency assessment should be completed?
3. Is there any evidence for the minimum number of hours of practice over a set period of time needed to maintain competency? Does this vary across professions or scope of practice?

## Methods

### Eligibility Criteria

Studies and reports will be included in the systematic review if they meet the following criteria:

1. The focus of the article or report is on CPD and/or ROP for health professions regulated in Australia
2. Reviews, original research, reports, and theses
3. For research question 4: reviews, original reports or theses that compare different types of CPD
4. Published from January 1, 2015, onward
5. Written in the English language

Articles and reports will be excluded from the review if they are:

1. Focused on health and other professionals not regulated under the National Law
2. Focused on students, interns, or residents
3. Focused on regulatory standards other than CPD and/or ROP
4. Opinion pieces, newsletters, and conference presentations
5. Published before January 1, 2015
6. Not written in the English language

### Information Sources

Databases to be searched for this review are as follows: the Allied and Complementary Medicine Database, MEDLINE, and PsycINFO (using the OVID platform), Better Evidence for Medical Education, CINAHL, the Campbell Collaboration of Systematic Reviews, the Cochrane Database of Systematic Reviews, Database of Abstracts and Reviews of Effects, Education Resources Information Centre, Embase, OTSeeker, Physiotherapy evidence, ProQuest Nursing and Allied Health, International Prospective Register of Systematic Reviews (PROSPERO), ScienceDirect, Web of Science, and Wiley Online Library.

These will be supplemented by hand searching relevant academic publications including but not limited to the *Aboriginal Health Worker*, *the Aboriginal and Islander Health Worker*, *Academic Medicine*, *the American Journal of Occupational Therapy*, *Australian Health Review*, *Australian Journal of Chiropractic*, *Australian Occupational Therapy Journal*, *BMC Medical Education*, *the British Medical Journal*, *Chiropractic Journal of Australia*, *Clinical Teacher*, *Compendium of Continuing Education in Dentistry*, *Education Journal of Dental Education*, *Journal of Alternative and Complementary Medicine*, *Journal of the American Medical Association*, *Journal of Chiropractic Education*, *Journal of Continuing Education in the Health Professions*, *Journal of Continuing Education in Nursing*, *Journal of Medical Internet Research*, *JMIR mHealth and uHealth*, *Journal of Medical Regulation*, *Journal of Nursing Regulation*, *Medical Education*, *the Medical Journal of Australia*, *Nurse Education in Practice*, *Nurse Education Today*, *Pharmacy Education*, *Physical Therapy*, *Prehospital Emergency Care*, and *Professional Psychology*.

Gray literature will be sourced from the websites for each of the national boards, relevant international health professional regulatory bodies (eg, Health and Care Professions Council, the United Kingdom), health professional associations (eg, Australian Podiatry Association, the Association of Canadian Occupational Therapy), relevant government departments (eg, Australian Government Department of Health).

Reference lists of articles and reports of interest will be hand searched, and a forward citation search will be conducted using Google Scholar and Web of Science.

### Search Strategy

Databases and other information sources will be searched for literature published between 2015 and 2021 in the English language. The search terms and sources of literature outlined below are based on our experience conducting a systematic review of the evidence for CPD and ROP standards for internal use based on journal articles and gray literature published between 1990 and 2014, and preliminary testing.

Medical Subject Headings (MeSH) by the National Library of Medicine will be used to search the databases outlined above, using all relevant root and hierarchical branches related to the terms. MeSH will also be explored to increase the ability to identify relevant publications where there are variations in the way articles are indexed. MeSH is a standardized hierarchically organized vocabulary developed by the National Library of Medicine to index, catalogue, and search biomedical- and health-related information.

MeSH terms related to health practitioner groups include “allied health occupations,” “acupuncture,” “chiropractic,” “dentistry,” “medicine,” “emergency medical technicians,” “medicine, traditional,” “midwifery,” “nurse-midwife,” “nursing,” “nursing, advanced practice,” “nursing practical,” “occupational therapists,” “optometry,” “osteopathic physicians,” “osteopathic medicine,” “pharmacy,” “physical therapists,” “physical therapy specialty,” “physicians,” “podiatry,” “psychology, medical,” and “radiologists.”

MeSH terms related to the intervention include “competency based education,” “education, continuing,” “education, distance,” “education, medical, continuing,” “education, nursing, continuing,” “education, pharmacy, continuing,” “learning,” “peer review, health care,” “return to work,” “self-assessment,” and “staff development.”

MeSH terms related to the outcome include “career mobility,” “competence, clinical,” “competence, professional,” “cultural competence,” “inappropriate prescribing,” “licensure,” “malpractice,” “mandatory programs,” “mandatory reporting,” “patient safety,” “problem behavior,” “professional practice,” “professionalism,” “quality of health care,” “risk management,” and “scope of practice.”

Additional search terms related to each health practitioner group include “Aboriginal and Torres Strait Islander health practitioner,” “Aboriginal and Torres Strait health worker,” “Chinese medicine practitioner,” “Chinese herbalist,” “chiropodist,” “medical radiation practitioner,” “nuclear medicine technologist,” “radiographers,” “radiotherapists,” “paramedics,” “physiotherapists,” “new graduate,” “early career,” “mature practitioner,” “mid-career,” “late career,” and “advanced practitioner.”

Additional search terms related to the intervention or event of interest include “accreditation,” “competency framework,” “competency standards,” “mentoring,” “objective structured clinical exam,” “on-line learning,” “practice portfolio,” “recency of practice,” “re-entry program,” “reflective practice,” “refresher program,” and “revalidation.”

Search terms related to outcomes include “advanced practice,” “authentic learning,” “endorsement,” “extended practice,” “fitness to practice,” “knowledge transfer,” “impaired practice,” “minimum practice hours,” “non-medical prescribing,” “registration standards,” “skills decay,” and “skills fade.”

As outlined in the PRISMA-P (Preferred Reporting Items for Systematic Reviews and Meta-Analyses Protocol) guidelines [2], an illustrative search is presented in Table 1 for one database.

**Table 1:** Example search of MEDLINE to identify literature on continuing medical education and professional competence in physicians (search conducted September 14, 2020).

Search number	Topic	Search field	Search term	Results, n
1	Health profession	MeSH^a^	exp Physicians/	142,586
2	Health profession	MeSH	limit 1 to yr= “2015-current”	39,403
3	Intervention	MeSH	exp Education, Medical, Continuing	24,857
4	Intervention	MeSH	limit 3 to yr= “2015-current”	3005
5	Outcome	MeSH	exp Competence, Clinical	93,723
6	Outcome	MeSH	limit 5 to yr= “2015-current”	24,417
7	Outcome	MeSH	2 and 4 and 6	218
8	Outcome	MeSH	limit 7 to English language	206

^a^MeSH: Medical Subject Headings.

### Study Records

#### Data Management

The search results will be imported into EndNote Software (version X9.3.3; Clarivate) [3], and duplicates will be removed using the Endnote “References/Find Duplicates” option. Full-text articles and reports will be stored in a secure location on our shared drive.

#### Selection Process

Titles listed in the search results will be checked, and the abstract will be consulted if the title appears relevant to any of the research questions. Articles and reports will be downloaded when the abstract gives the impression of being pertinent to the research questions and checked for inclusion in the review. As noted above, inclusion or exclusion and, where applicable, the reasons for exclusion, will be recorded.

#### Data Collection Process and Data Items

A Microsoft Excel (Microsoft Corp) spreadsheet will be used to record bibliographic information about each article or report (eg, author, date, title), the study population (eg, health profession, size, country), intervention (eg, type of CPD), main findings, study type, National Health and Medical Research Council level of evidence [4], decisions as to inclusion or exclusion (including any reasons for exclusion), and the quality assessment.

### Quality Appraisal

Where the full text of the article is assessed as relevant to the research questions, quality appraisal will be conducted by 2 people using the weighted evidence approach developed by the Evidence for Policy and Practice Information and Co-ordinating Centre at the Institute for Education in the University of London [5].

Briefly, this method provides an overall score to be derived for each study by assigning a score (high=1, medium=2, low=3) against each of the 3 criteria listed below and summing the scores.

1. The trustworthiness of the results judged by the quality of the study within the accepted norms for the particular research design used in the study (methodological quality)
2. The appropriateness of the study design for addressing the systematic review’s research question (methodological relevance)
3. The appropriateness of the focus of the research for answering the review question (topic relevance)

The overall rating is derived by summing the scores assigned for each of the criteria. The overall weight of evidence would therefore be indicated as high (3,4), medium (5,6), or low (7-9).

Two reviewers will independently assess the weight of evidence of the included studies, and their assessment will be recorded on the spreadsheet.

### Data Extraction and Reporting

Data extraction and quality assessment will be undertaken by the primary reviewer (PM). A second reviewer (SA) will confirm the accuracy of the data. Any disagreements will be resolved through discussion or third-party adjudication. Data extraction by a single reviewer results in considerable time saving and has little impact on the conclusions [6].

As meta-analysis is not feasible for this type of systematic review, the findings will be reported in narrative form with information about the included studies presented in tabular form and published in a peer-reviewed journal. The narrative will draw out the main themes of the systematic review and discuss their implications in the context of health practitioner regulation in Australia.

## Results

### Studies Relevant to the CPD Research Questions

In September 2021, our search strategy identified 18,791 studies through database searching, with an additional 96 records identified through other sources, resulting in 18,002 records after duplicates were removed. Of these, 509 records were screened based on their title, and 17,493 records were excluded. A total of 66 full-text articles were assessed for eligibility based on their abstract, of which 35 full-text articles were excluded because they did not meet the inclusion criteria. A total of 31 studies met the inclusion criteria.

### Studies Relevant to the ROP Research Questions

Our search strategy identified 278 studies through database searching, with an additional 25 records identified through other sources, resulting in 291 records after duplicates were removed. Of these, 87 records were screened based on their title and 41 records were excluded. Forty-six full-text articles were assessed for eligibility based on their abstract, of which 38 were excluded, leaving 8 studies that met our inclusion criteria.

## Discussion

This is the first systematic review completed by Ahpra and the National Boards of the evidence for CPD and ROP standards that covers all health professions regulated by the National Scheme. As such, its focus is wider than that of the recently published protocol for a scoping review of ROP for nurses and midwives [7].

It is anticipated that this systematic review will provide a comprehensive evidence base for CPD and ROP requirements for professions regulated by Ahpra. Previous research has found that even though there is good evidence to show CPD is effective in increasing practitioner knowledge, there is less evidence supporting that CPD changes clinical practice, and even less evidence linking CPD to improved patient safety [8-10]. In 2010, an Institute of Medicine study in Washington found major flaws in the way in which CPD was conducted, financed, regulated, and evaluated [11], which led to a greater focus on strategies such as maintenance of certification and other quality assurance activities [12-15]. A CPD mapping exercise conducted in 2015 found considerable variance in CPD standards across European jurisdictions, with a trend toward increased mandatory requirements for CPD and revalidation [16]. ROP was found to be a poorly researched area with most research concentrating on medical practitioners, nurses, and midwives, with no clear consensus about the optimal time period after which retraining or an assessment of competence should be introduced [17,18].

This protocol has been designed to identify, summarize, and assess the quality of the evidence published to date for CPD and ROP registration standards for selected regulated health professions in Australia. As outlined above, a major strength of the method is that it covers a broad range of health professions. Other strengths include research questions that are designed to support the planned review of CPD and ROP standards in Australia, a comprehensive search strategy with clearly defined inclusion and exclusion criteria, and an appropriate instrument to assess the quality of the evidence for use in the development of public policy.

Potential limitations of the method include the following: there are differences in standards for health practitioners in jurisdictions where publications are found and those in Australia; there are likely to be fewer publications focusing on professions with lower numbers of registrants; and, due to the nature of the review, the authors are unable to correct study biases.

In conclusion, this protocol describes a detailed method for a systematic review of the evidence for CPD and ROP registration standards for health practitioners. This review will inform a multiprofession review of CPD and ROP standards in Australia. The findings will be of interest to regulators of health practitioners in other jurisdictions and may be used to inform international regulatory standards.

## Abbreviations

Ahpra: Australian Health Practitioner Regulation Agency
CPD: continuing professional development
MeSH: Medical Subject Headings
National Scheme: National Registration and Accreditation Scheme
PRISMA-P: Preferred Reporting Items for Systematic Reviews and Meta-Analyses Protocol
PROSPERO: International Prospective Register of Systematic Reviews
ROP: recency of practice
